# Advancing Non-Invasive Ophthalmic Imaging in Sturge–Weber Syndrome: Clinical Guidelines Towards Early Choroidal Hemangioma Detection

**DOI:** 10.3390/jcm14197012

**Published:** 2025-10-03

**Authors:** Mariachiara Di Pippo, Daria Rullo, Chiara Ciancimino, Flaminia Grassi, Alessandro Ferretti, Pasquale Parisi, Giovanni Di Nardo, Alessandro Orsini, Marco Perulli, Domenica Immacolata Battaglia, Ezio Maria Nicodemi, Solmaz Abdolrahimzadeh

**Affiliations:** 1 Ophthalmology Unit, Neurosciences, Mental Health, and Sense Organs (NESMOS) Department, Faculty of Medicine and Psychology, University of Rome Sapienza, 00135 Rome, Italy; mariachiara.dipippo@uniroma1.it (M.D.P.); daria.rullo@uniroma1.it (D.R.); chiara.ciancimino@uniroma1.it (C.C.); flaminiagrassi@gmail.com (F.G.); 2 Pediatrics Unit, Neuroscience, Mental Health and Sense Organs (NESMOS) Department, Faculty of Medicine and Psychology, Sapienza University of Rome, 00135 Rome, Italy; alessandro.ferretti@uniroma1.it (A.F.); pasquale.parisi@uniroma1.it (P.P.); giovanni.dinardo@uniroma1.it (G.D.N.); 3 St. Andrea Hospital, 00189 Rome, Italy; 4 Pediatric University Department, Azienda Ospedaliera Universitaria Pisana, University of Pisa, 56121 Pisa, Italy; aorsini.md@gmail.com; 5 Department of Life Sciences and Public Health, Università Cattolica del Sacro Cuore, 00168 Rome, Italy; doc.perulli@gmail.com (M.P.); domenicaimmacolata.battaglia@policlinicogemelli.it (D.I.B.); 6 Child Neurology and Psychiatric Unit, Fondazione Policlinico Universitario Agostino Gemelli, IRCCS, 00168 Rome, Italy; 7 IRCCS IDI—FLMM, 00167 Rome, Italy; e.nicodemi@idi.it

**Keywords:** Sturge Weber syndrome, diffuse choroidal hemangioma, retinal imaging, enhanced depth spectral domain optical coherence tomography, multimodal imaging

## Abstract

**Background/Objectives**: Sturge–Weber syndrome (SWS) is a rare neuro-oculocutaneous disorder characterized by leptomeningeal angioma, naevus flammeus, and ocular manifestations, including diffuse choroidal hemangioma (DCH). This study compares the diagnostic performance of near-infrared reflectance (NIR) imaging and enhanced depth imaging spectral-domain optical coherence tomography (EDI-SDOCT) with fundus photography in detecting DCH. **Methods**: Seventeen patients with SWS underwent comprehensive ophthalmologic evaluation, including fundus photography, NIR, and EDI-SDOCT imaging. Sensitivity, specificity, and accuracy of fundus photography, NIR, and EDI-SDOCT were calculated. **Results**: Sixteen patients had evaluable data. DCH was identified by fundus photography in five (31%), NIR in three (18.75%), and EDI-SDOCT in fourteen patients (87.50%). EDI-SDOCT alone demonstrated 100% sensitivity and 100% accuracy, outperforming both NIR (21.4% sensitivity; 31.6% accuracy) and fundus photography (35.7% sensitivity; 43.8% accuracy). When positive findings on NIR and/or SDOCT were combined, sensitivity and accuracy reached 100%. EDI-SDOCT provided detailed morphologic visualization of the choroid, allowing for early diagnosis of DCH even in pediatric cases with limited patient cooperation. **Conclusions**: EDI-SDOCT significantly improves the detection of DCH in SWS compared with fundus photography and NIR. Given its superior sensitivity and accuracy, incorporating EDI-SDOCT into routine clinical assessment may enable earlier diagnosis and reduce retinal complications in SWS.

## 1. Introduction

Sturge–Weber syndrome (SWS) is a sporadic neuro-oculocutaneous condition with a wide variety of cutaneous, neurological, and ocular manifestations. The prevalence of SWS is approximately 1 in 20,000 to 50,000 live births [1]. The somatic activating mutation in the GNAQ gene has been shown to cause SWS, and partial manifestations of the syndrome are due to the late origin of the mutation in endothelial cells [2]. Ocular involvement occurs in 50% to 70% of cases, including glaucoma and vascular formations of the periorbital skin, conjunctiva, sclera, and choroid [3].

The most frequent cutaneous vascular feature, present at birth, is the facial nevus flammeus [4]. Neurological signs include seizures, stroke-like episodes, developmental delay, and headache [5,6]. Neuroradiologically, leptomeningeal angiomatosis can be identified, but early signs include vascular and parenchymal abnormalities [3,7]. The two most common ophthalmological conditions include glaucoma in 30–70% of cases and diffuse choroidal hemangioma (DCH) in approximately 50% of cases, usually ipsilateral to the facial naevus flammeus [3].

From a genetic perspective, SWS, arises from somatic mosaic activating mutations in the *GNAQ* gene. Shirley et al. first identified the recurrent *GNAQ* p.R183Q substitution in affected skin and brain tissue from patients with SWS and in non-syndromic port-wine stains, establishing this mutation as the molecular hallmark of the disease [2]. Subsequent studies confirmed that *GNAQ* R183Q is mutated in endothelial cells, forming capillary malformations and leptomeningeal or ocular lesions [8]. More recently, Francis et al. demonstrated that DCH consistently harbors *GNAQ* R183Q, whereas solitary circumscribed hemangiomas carry *GNAQ* Q209 mutations, similar to those found in uveal melanoma. Together, these findings highlight that *GNAQ* R183Q is associated with diffuse vascular malformations such as those seen in SWS, whereas Q209 variants are linked to solitary, tumor-like ocular lesions, underscoring a distinct genotype–phenotype correlation [9].

In clinical practice, slit lamp examination facilitates the assessment of anterior segment vascular alterations, and glaucoma is usually diagnosed early in the course of the disease. However, DCH is difficult to assess with ophthalmoscopic examination, and it is often diagnosed if retinal complications arise. Most published articles describe DCH as a “tomato ketchup” coloration on dilated fundus examination [3]; however, this appearance can easily be overlooked in routine ophthalmoscopic assessment. Thus, fundus photography is often necessary for comparison between eyes, especially in children who have difficulty cooperating.

Imaging methods to detect DCH are ultrasonography, indocyanine green angiography (ICGA), fundus fluorescein angiography (FFA), and magnetic resonance imaging [10]. However, these methods are invasive, the use of a contrast medium increases risk, and substantial patient collaboration is required; thus, they are difficult to carry out in children. Furthermore, ocular ultrasonography may be uncomfortable in pediatric and young patients, since it requires direct contact with the ocular surface and the application of gel, often causing discomfort and limiting cooperation. Currently, non-invasive multimodal imaging methods such as spectral-domain optical coherence tomography (SDOCT) and near-infrared reflectance (NIR) have entered routine clinical practice with a paradigm shift in the diagnosis of choroidal and retinal pathologies. Indeed, enhanced depth imaging (EDI) SDOCT can enable early diagnosis of DCH [11,12]. Swept-source OCT instruments enable a better definition of the choroid [10]; however, this instrument is not readily available in all practices.

The recently published consensus statement on SWS indicates referral to a pediatric ophthalmologist in any child presenting a facial naevus flammeus [7]. A high-risk naevus flammeus, indicating the possible ipsilateral development of glaucoma in childhood, is characterized as extending from the forehead to the midline between the superior section of the ear and the outer cantus, including the upper eyelid [4,13]. The diagnostic workup for glaucoma in SWS is relatively straightforward, but ophthalmologists lack clinical guidelines for evaluating choroidal alterations. Most data in the literature are based on case reports and small case series owing to the rare nature of the disease; thus, there is a current need for an established clinical diagnostic workup for DCH.

While the primary focus of research has been on diagnosis, a wide range of treatment modalities has also been investigated in the literature, including photodynamic therapy (PDT) [14,15], brachytherapy with I-125 or Ru-106 plaques [16,17,18], proton beam and intensity-modulated radiotherapy [19], as well as pharmacological approaches such as propranolol [20,21] and intravitreal anti-VEGF agents [22,23]. These strategies, each with distinct efficacy and safety profiles, highlight the need for individualized management of DCH.

In this report, we aimed to provide evidence-based guidelines for clinical practice in diagnosing DCH in patients with SWS using non-invasive multimodal imaging. In particular, the primary endpoint was to compare the diagnostic efficacy of SDOCT and NIR imaging with respect to fundus photography by assessing sensitivity, specificity, and diagnostic accuracy.

## 2. Materials and Methods

This was a diagnostic translational accuracy study in which seventeen patients with a confirmed diagnosis of SWS recruited at the Retina Center of the Ophthalmology Unit, Department of Neurosciences, Mental Health and Sensory Organs (NESMOS), Sapienza University of Rome, St. Andrea Hospital, were enrolled. Written informed consent was obtained from each patient or, in the case of minors, from a parent or guardian. The study was conducted in accordance with the tenets of the Declaration of Helsinki.

Diagnosis of SWS was confirmed based on the presence of leptomeningeal angioma, a naevus flammeus in the trigeminal nerve area distribution, and ocular involvement (glaucoma and/or DCH). Patients meeting all three criteria were classified as having classic SWS, whereas those with two criteria were classified as having the fruste form. Inclusion criteria were the presence of two or three of the diagnostic criteria, sufficient patient cooperation for visual acuity and imaging procedures, and clear optical media for imaging. Exclusion criteria included any systemic or ocular disease, other pathologies associated with SWS, and ocular surgery within the preceding six months. Demographic characteristics of the patients are reported in Table 1.

All eligible subjects underwent a general, ocular, and pharmacological history assessment; best-corrected visual acuity measurement; and anterior segment evaluation, including intraocular pressure measurement and photographic documentation of the facial naevus flammeus and anterior segment structures at the slit lamp. A fundus examination was performed following pupillary dilation with 1% tropicamide, in accordance with routine clinical practice. Fundus photographs were obtained using the integrated photographic module of the Solix device (Solix, Optovue, CA, USA).

NIR imaging and SDOCT were performed with the EDI mode (Spectralis OCT+HRA, Heidelberg Engineering, Heidelberg, Germany). Fovea-centered scans were obtained using a raster protocol or line scan mode according to the DCH extension, depth and patient collaboration, with 100 frames averaged per scan. OCT scans with acquisition difficulties (e.g., poor fixation, motion artifacts, or inadequate signal) were excluded from analysis. Only images with acceptable quality were retained, defined as a minimum Heidelberg Spectralis OCT quality score (Q-score) of ≥20 [24,25].

Three independent retinal imaging experts evaluated the fundus photographs, NIR images, and EDI-SDOCT scans for the presence of DCH. The experts were not blinded to the diagnosis of SWS. All three retinal imaging experts were in full agreement regarding the presence of DCH. Inter-rater reliability analysis demonstrated high consistency across modalities. Overall agreement was excellent (Fleiss’ κ = 0.84; 95% CI, 0.76–0.92). By modality, agreement was substantial for fundus photographs (κ = 0.78; 95% CI, 0.66–0.90), excellent for NIR images (κ = 0.86; 95% CI, 0.78–0.94), and almost perfect for EDI-SDOCT scans (κ = 0.91; 95% CI, 0.84–0.98).

**Figure 1:**
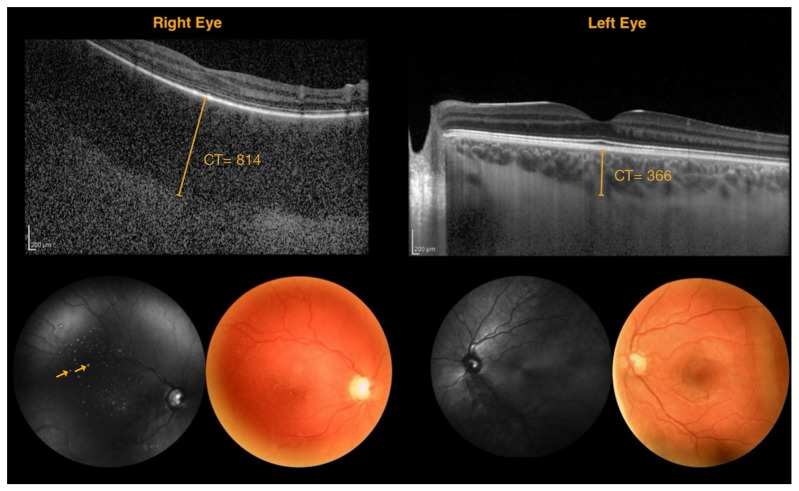
Multimodal imaging in a patient with diffuse choroidal hemangioma of the right eye. Right eye: spectral-domain optical coherence tomography scan shows diffuse choroidal thickening and pseudo-drusen-like lesions; fundus photography shows the absence of tessellation, vascular tortuosity, drusen-like lesions, macular hyperpigmentation, and a large optic disk/cup ratio; near-infrared reflectance image shows hyperreflective dots (arrows) and vessel alterations. (CT = choroidal thickness measured in μm).

On fundus photography, DCH was defined as the presence of an area of hypopigmentation or irregular choroidal vasculature in the posterior pole, often appearing as a tomato ketchup-colored area with blurred margins. On NIR imaging, the presence of hyperreflective dots within a hyperreflective area or hyperreflective dots with respect to the fellow eye was an encountered feature of DCH. On EDI-SDOCT, DCH was identified as an increase in choroidal thickness with respect to the fellow eye. Subfoveal choroidal thickness was measured from the inferior margin of Bruch’s membrane to the choroidoscleral junction. Additionally, alterations in the reflectivity of the choroidal vascular structural pattern were assessed, considering differences with respect to surrounding regions and the fellow eye. Finally, the diagnostic performance of each imaging modality in detecting DCH was evaluated. Figure 1 shows an example of multimodal imaging in a patient with a DCH in the right eye.

SWS is a very rare disease, so we could not perform a conventional sample size calculation. We enrolled about 18 patients with SWS. This sample size allowed for estimating sensitivity with a 10% margin of error and a 95% confidence interval, assuming a 95% sensitivity and a 60% prevalence of choroidal hemangioma. It also enabled the evaluation of concordance with an 11% margin of error and a 95% confidence interval, assuming a Cohen’s kappa of 0.95.

Quantitative data were presented as mean and standard deviation (SD) and as median and first and third quartiles (Q1–Q3). Categorical data were presented as numerical values and percentages (%). When necessary, in the analysis, the presence of multiple data points in the same subject (two eyes) was accounted for with the use of mixed-effects models. Inter-rater reliability among the three retinal imaging experts was assessed using Fleiss’ kappa for categorical presence/absence determinations, with 95% confidence intervals calculated using the standard error method. A *p*-value of <0.05 was considered statistically significant.

## 3. Results

Eighteen patients were screened for the study; one patient was excluded owing to poor collaboration due to disease-correlated intellectual disability (patient 6), while one patient was excluded as the baseline visit occurred beyond the enrollment phase cut-off (patient 18).

Data were therefore collected from 17 patients to 7 males (41%) and 10 females (59%)—ranging in age from 4 to 68 years, with a mean (SD) age of 28 (20.7) and a median (Q1–Q3) age of 20 (12–45). Table 2 summarizes the morphological characteristics identified using the multimodal imaging modalities.

The primary outcome could only be evaluated in 16 patients: seven males (44%) and nine females (56%), ranging in age from 4 to 68 years, with a mean (SD) age of 29 (21.0) years and a median (Q1–Q3) age of 20 (12–47) years. NIR imaging yielded a positive diagnosis of DCH in 3 patients (18.75%), whereas EDI-SDOCT was positive in 14 patients (87.50%). Two patients were negative for DCH on both NIR and EDI-SDOCT, three were positive on both NIR and EDI-SDOCT, and eleven were positive on EDI-SDOCT but negative on NIR.

Using fundus photography, five of sixteen patients (31%) were found to have DCH. Overall, two patients were negative on all imaging modalities, whereas two were positive on all. Fundus photography demonstrated a sensitivity of 35.7% (5/14) and an accuracy of 43.8% (7/16). However, when considering patients positive if they tested positive on either NIR or SDOCT, the combined NIR + EDI-SDOCT approach achieved a sensitivity of 100% and an accuracy of 100%. Evaluating these methods separately, NIR alone showed a sensitivity of 21.4% (3/14) and an accuracy of 31.6% (5/16), whereas EDI-SDOCT alone demonstrated a sensitivity of 100% and an accuracy of 100%. No significant adverse events occurred as a result of fundus photography, NIR, or EDI-SDOCT imaging.

As a secondary objective, choroidal thickness was measured in all 17 patients; however, reliable values in both eyes were obtained in only 12 cases. Table 3 reports the choroidal thickness of the affected and fellow eyes, with ‘NA’ indicating cases where measurements were not feasible. In the 12 patients with bilateral data, interocular differences ranged from −14 μm to 540 μm, with a consistent trend toward greater choroidal thickness in eyes affected by DCH compared to their fellow eyes.

## 4. Discussion

The present study showed the diagnostic efficacy of multimodal imaging techniques, particularly EDI-SDOCT and NIR, in the detection of DCH in patients with SWS. EDI-SDOCT significantly outperformed fundus photography and NIR imaging, serving as an accurate and non-invasive diagnostic tool that is particularly useful in pediatric populations with limited cooperation.

Choroidal hemangiomas are benign vascular tumors that most commonly affect choroids. These lesions can be classified as either circumscribed or diffuse. The latter subtype accounts for approximately 50% of all choroidal hemangiomas and is typically associated with SWS [26,27,28]. Histologically, choroidal hemangiomas are hamartomas and are characterized by small or large blood vessels surrounded by varying degrees of loose connective tissue [26]. Although these formations are benign and lack invasive properties, they can exert significant mechanical pressure on overlying structures, leading to complications such as neurosensory retinal detachment, retinal pigment epithelium (RPE) elevation, RPE pigmentation changes, and subretinal fibrosis [26,27,28,29]. These complications often emerge at advanced stages of the disease, coinciding with the onset of visual impairment, which frequently leads to diagnosis. DCH has traditionally been identified through fundoscopic examination and described in the literature as indistinct red-orange lesions with poorly demarcated margins, imparting a “tomato ketchup” appearance to the fundus [26,27,30,31,32]. In most cases, these lesions are unilateral, a feature that aids diagnosis by allowing color comparison with the unaffected fellow eye. However, fundoscopic diagnosis poses significant challenges, particularly in pediatric patients, as it is highly operator-dependent, non-reproducible, and does not allow for longitudinal comparison. To address these limitations, fundus photography is used to facilitate longitudinal monitoring and improve lesion assessment relative to the fellow eye. Despite these advantages, fundus photography remains constrained by the subtlety of the clinical findings and the potential for suboptimal image quality due to poor patient cooperation.

Our results confirmed that the detection of DCH using fundus photography is limited due to its subjective nature and dependence on observer experience, demonstrating a sensitivity of 35.7% and an accuracy of 43.8%. The classic “tomato ketchup” appearance may be easily overlooked, particularly in cases without a direct comparison with the fellow eye. Surve et al. reported choroidal vascular patterns in patients with SWS using swept-source OCT (SSOCT) compared to fundus photography, FFA, and ICGA. Their findings showed that DCH detection rates were 50% with fundus photography, 52.94% with FFA, 82.35% with ICGA, and 86.36% with SSOCT [10].

FFA and ICGA are additional retinal imaging modalities used for diagnosing DCH; however, their standalone effectiveness in identifying DCH remains controversial. FFA, which primarily enables the assessment of the retinal circulation, may not reliably confirm the presence of DCH in its early stages but highlights complications such as neurosensory detachment or RPE fibrotic changes [31,33]. ICGA, which specifically examines the choroidal circulation, may be more sensitive, showing an early hyperfluorescence of the vascular network within the hemangioma, which increases in intensity in intermediate phases before exhibiting marked hypofluorescence in the late phases, a phenomenon known as wash out [31,33,34]. This superiority of ICGA was reported by Surve et al. (52.94% with FFA versus 82.35% with ICGA). However, FFA and ICGA are invasive techniques requiring dye administration, are costly, and require substantial patient cooperation, making early-stage DCH detection challenging, particularly in children [10].

SDOCT is a non-invasive imaging modality that provides high-resolution visualization of retinal and choroidal structures. In particular, the EDI technique shifts the zero-delay plane posteriorly to the RPE, thus enhancing signal sensitivity and reducing noise, allowing for detailed choroidal imaging. This technique is currently employed for diagnosing and monitoring choroidal structure alterations in various retinal and systemic pathologies [19,20]. EDI-SDOCT allows both qualitative and quantitative assessment of DCH. Qualitatively, a DCH appears as an alteration in choroidal vascular reflectivity, typically presenting as a hyporeflective lesion that may extend and affect retinal and surrounding choroidal structures [31]. Quantitatively, high-resolution imaging with EDI-SDOCT allows for precise evaluation and comparison of choroidal thickness with respect to the fellow eye [11,12]. This enables diagnosis even in cases where choroidal vascular morphology remains relatively preserved but the DCH manifests as a generalized choroidal thickening. Additionally, EDI-SDOCT enables the identification of associated DCH complications such as Bruch’s membrane rupture, RPE alterations, and neurosensory detachment with subretinal fluid accumulation [35]. Choroidal thickness measurements were attempted in all 17 patients; however, reliable values in both eyes were obtained in only 12 cases. In the 12 patients with bilateral data, interocular differences ranged from -14 μm to 540 μm, with a consistent trend toward greater thickness in eyes with DCH. However, choroidal thickness values were highly variable across patients, reflecting the well-established physiological variability of this parameter. Indeed, choroidal thickness has been shown to decrease with age and to undergo significant diurnal fluctuations, which complicates the establishment of standardized reference values [36,37].

**Figure 2:**
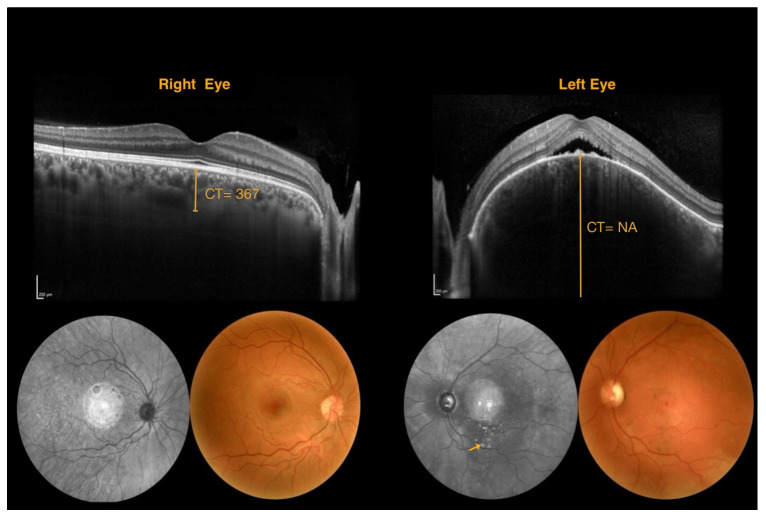
Multimodal imaging for a patient with diffuse choroidal hemangioma of the left eye. Left eye: spectral-domain optical coherence tomography scan shows a choroidal lesion, neuroepithelium detachment with associated subretinal fluid, and pseudodrusen-like lesions; fundus photography shows the absence of tessellation, vascular tortuosity, drusen-like lesions, and macular hyperpigmentation, near-infrared reflectance imaging presents hyperreflective dots (arrow) and vessel alterations. Choroidal thickness is not measurable in the left eye as the choroidoscleral junction is not visible. (CT = choroidal thickness measured in μm. NA = not applicable).

In our study, we aimed to complement EDI-SDOCT with NIR imaging, a fast and non-invasive technique that can be easily used in young patients [38]. Notably, previous case reports demonstrated the presence of hyperreflective punctate lesions on NIR imaging within hyporeflective choroidal regions, coinciding with the appearance of micro-drusen-like lesions on SDOCT scans [39]. Our study identified hyperreflective punctate lesions in 11 eyes (Figure 2). However, in only a subset of these cases could these lesions be definitively associated with a DCH diagnosis. Consequently, NIR imaging alone demonstrated a sensitivity of 21.4% (3/14 eyes) and an accuracy of 31.6% (5/16 eyes), further emphasizing a multimodal imaging approach for accurate DCH detection. In contrast, EDI-SDOCT demonstrated superior sensitivity (100%) and accuracy (100%), making it the most reliable method for DCH diagnosis.

**Figure 3:**
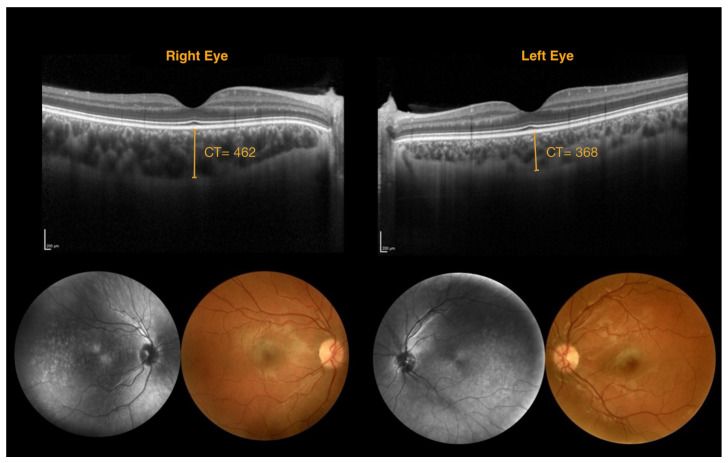
Multimodal imaging for a patient with diffuse choroidal hemangioma of the right eye. Fundus photography and near-infrared reflectance imaging do not reveal any significant characteristic alterations. However, the spectral-domain optical coherence tomography scan shows an increase in choroidal thickness in the right eye compared to the fellow eye. (CT = choroidal thickness measured in μm).

These findings align with a previous report in the literature that emphasized the importance of advanced imaging techniques in diagnosing choroidal involvement in SWS [10]. Though effective, traditional methods, including ultrasonography and dye-based angiographic techniques, have limitations due to their invasive nature, cost, and need for patient cooperation. SSOCT is a valid alternative and offers better choroidal visualization but has limited availability in routine clinical settings [10]. Our study identifies EDI-SDOCT as an accessible and effective diagnostic modality for widespread use in ophthalmology. Figure 3 shows DCH diagnosed with EDI-SDOCT in a case where fundus photography yielded a negative result.

In clinical practice, the early and accurate diagnosis of DCH is critical for preventing retinal complications such as subretinal fluid (SRF) formation, neurosensory retinal detachment, retinal pigment epithelium (RPE) elevation, RPE pigmentation changes, and subretinal fibrosis [26,27,28,29]. From a therapeutic standpoint, several strategies have been investigated to manage symptomatic DCH. Photodynamic therapy (PDT) achieves meaningful anatomic and functional gains, but carries risks of choroidal atrophy and fibrosis in the long term and requires careful retreatment planning [14,15]. Episcleral plaque brachytherapy (I-125/Ru-106) is effective for both circumscribed and diffuse hemangiomas; intra-operative ultrasound guidance improves targeting under bullous retinal detachment, enabling regression and SRF with globe preservation [16,17,40]. Recent pediatric data show I-125 apex doses around 34–42 Gy achieving durable tumor control, with reduced radiation complications such as cataract and subretinal fibrosis [18].

For diffuse diseases involving the entire choroid, external beam radiation therapy (EBRT) has historically been employed, with high rates of exudative retinal detachment resolution but also significant collateral damage, including cataract, orbital pain, and optic neuropathy, which limited its widespread adoption. Intensity-modulated radiation therapy (IMRT) represents a refinement of EBRT, using non-uniform, lens-sparing beams shaped by multileaf collimators to deliver a low total dose (≈20 Gy in 10 fractions). This approach achieves nearly universal SRF resolution and significant tumor regression/stability, while minimizing collateral toxicity. Long-term series confirm durable efficacy with no recurrences and an excellent safety profile, making IMRT one of the most promising options for DCH management [19]. Proton beam radiation therapy (PBRT) similarly allows highly uniform dose delivery to the choroid and has shown high rates of tumor regression and SRF resolution. However, PBRT may be associated with a higher risk of cataract, radiation optic neuropathy, and macular edema in some cohorts, requiring careful patient selection and long-term monitoring. Taken together, EBRT, IMRT and PBRT expand the therapeutic armamentarium beyond focal treatments, offering reliable globe- and vision-preserving results in extensive DCH. Adjunctive pharmacologic approaches include anti-VEGF therapy in cases with choroidal neovascularization or macular edema [22,23], and systemic propranolol, which has demonstrated variable efficacy in resolving subretinal fluid and lowering IOP in SWS patients with glaucoma [20,21].

As our study demonstrated, fundus photography alone is insufficient for diagnosis, necessitating a shift towards EDI-SDOCT and multimodal imaging for screening of SWS patients. Thus, we propose that ophthalmologists incorporate SDOCT into standard diagnostic workflows for patients with facial naevus flammeus, particularly those with a history of glaucoma and DCH. This evidence supports the routine use of SDOCT for detecting DCH in patients with SWS. Given its high sensitivity and accuracy, EDI-SDOCT could be considered the gold standard for diagnosing choroidal involvement in SWS, complementing traditional clinical assessment and fundus photography. This is particularly relevant in young patient populations, where non-invasive and fast imaging modalities are preferred, and where early diagnosis may help prevent vision-threatening complications. Moving forward, OCT angiography (OCTA) and other emerging imaging modalities hold promise for further advancing the assessment of DCH. OCTA could provide qualitative and quantitative data on the vascular characteristics of DCH, complementing structural SDOCT findings and potentially yielding biomarkers for disease activity or progression. Combining high-resolution imaging techniques may further refine diagnostic algorithms and contribute to further research in the field.

### Limitations of the Study

The limitations of our study include the small sample size, which is largely attributable to the rarity of SWS and DCH, limiting the generalizability of our findings. Another limitation is the absence of a dedicated healthy control group for the evaluation of choroidal thickness symmetry. Some choroidal thickness measurements were not available due to technical difficulties and collaboration issues (as for patients 1 and 6), and this could be a potential limitation. However, the purpose of the study was a comparison of the diagnostic efficacy of SDOCT and NIR imaging with respect to fundus photography and not a strict measurement of choroidal thickness.

A further limitation of the present study is the absence of an external control group. However, this was considered methodologically appropriate, as choroidal thickness lacks universally established normative reference values and is known to vary substantially with age and according to diurnal fluctuations [36,37]. For these reasons, the fellow eye was deemed the most reliable internal comparator in the evaluation of interocular differences.” Moreover, automated choroidal segmentation is not available on the SDOCT instrument or the software that we used. Although new swept-source OCT devices provide this possibility, such instruments are not widely available in routine practice. These factors, together with methodological considerations, should be considered when interpreting our findings. Future studies with larger multicentre cohorts, improved image acquisition, and inclusion of a control group will be valuable to confirm and extend our results.

## 5. Conclusions

This study demonstrates that EDI-SDOCT is a highly sensitive, accurate, and non-invasive tool for the detection of DCH in patients with SWS, outperforming traditional fundus photography and NIR imaging. The ease of use and reproducibility of EDI-SDOCT make it particularly suitable for routine clinical practice, especially in pediatric populations, where cooperation can be limited. We propose that EDI-SDOCT be integrated into standard ophthalmologic workflows for patients with facial naevus flammeus or suspected SWS, enabling earlier detection of choroidal involvement, timely monitoring, and more informed clinical decision-making. Routine use of this modality may ultimately help prevent vision-threatening complications and guide patient management, while multimodal imaging, including NIR and future OCTA, can further enhance diagnostic precision. Larger studies are warranted to validate these findings and to establish standardized imaging protocols for pediatric ophthalmology practice.

## Figures and Tables

**Table 1:** Demographic and ocular characteristics of patients with Sturge–Weber syndrome.

Patient Number	Age	Sex	Leptomeningeal Angioma	Nevus Flammeus	Glaucoma	BCVA (ETDRS Charts)		IOP RE/LE	Enrolled
						RE	LE		
1	27	F	YES	YES (LE)	YES (LE)	25	0	12/13	YES
2	16	M	YES	YES (LE)	YES (LE)	47	48	14/10	YES
3	42	F	NO	YES (RE)	YES (RE)	0	57	20/12	YES
4	20	M	NO	YES (RE)	YES (RE)	27	51	18/16	YES
5	48	F	YES	YES (RE)	YES (RE)	52	55	17/14	YES
6	13	F	YES	YES (RE)	YES (RE)	NA	NA	NA	NO
7	19	M	NO	YES (RE)	YES (RE)	48	51	20/16	YES
8	4	M	YES	YES (RE)	NO	55	55	12/12	YES
9	7	M	YES	YES (RE)	YES (RE)	0	35	14/16	YES
10	17	F	YES	YES (RE)	YES (RE)	0	35	14/16	YES
11	38	F	YES	YES (LE)	NO	55	50	10/17	YES
12	20	F	NO	YES	YES	5	53	18/14	YES
13	68	M	NO	YES (LE)	YES (LE)	55	0	14/20	YES
14	4	F	YES	YES (RE)	NO	NA	NA	12/12	YES
15	57	F	NO	YES (LE)	NO	55	55	14/14	YES
16	62	F	NO	YES (RE)	NO	55	55	14/14	YES
17	11	M	YES	YES (RE)	NO	53	55	18/18	YES

BCVA (best-corrected visual acuity); ETDRS (Early Treatment Diabetic Retinopathy Study); RE (Right eye); LE (Left eye); IOP (Intraocular pressure in therapy in the eye involved); NA (not applicable).

**Table 2:** Near-infrared reflectance, fundus photography, and spectral-domain optical coherence tomography findings in Sturge–Weber syndrome. “Yes” indicates that the modality alone was sufficient to detect DCH, while “No” indicates that the modality did not provide sufficient evidence for a DCH diagnosis. “NA” denotes that imaging with the respective modality was not available due to limited patient cooperation. The table illustrates the comparative diagnostic performance of the three imaging techniques.

Patient Number	Diagnosis Through Fundus Photography	Fundus Photography Findings	Diagnosis Through SDOCT	SDOCT Findings	Diagnosis Through NIR	NIR Findings
1	NO, poor quality imaging	NA	YES	LE: hyporreflective choroidal lesion associated with paralesional choroidal folds	NO, poor quality imaging	demarcation line
2	YES	chorioretinal folds, no tessellation, arteriovenous crossing	YES	LE: hyporreflective choroidal lesion, inner retina folding, paralesional choroidal fold	NO	hyperreflective area/dots (temporal), vessel alterations
3	NO	RE chorioretinal folds	YES	RE: hyporreflective choroidal lesion, neuroepithelium detachment with associated subretinal fluid	YES	lacquer cracks, hypo-hyperreflectivity
4	NO	RE chorioretinal folds	YES	RE: hyporreflective choroidal lesion	NO	folds, diffuse hypo-hyperreflectivity
5	YES	no tessellation	YES	RE: choroidal thickening	NO	hyperreflective area/dots (temporal)
6	NA	NA	NA	NA	NA	NA
7	YES	no tessellation, vascular tortuosity, chorioretinal folds	YES	RE: hyporreflective choroidal lesion	YES	hyperreflective area/dots (temporal), vessel alterations
8	NO	NA	YES	RE: diffuse choroidal thickening	NO	hyperreflective area/dots (temporal)
9	YES	no tessellation, vascular tortuosity	YES	RE: diffuse choroidal thickening	NO	NA
10	YES	no tessellation, vascular tortuosity, drusen-like lesions, macular hyperpigmentation	YES	RE: diffuse choroidal thickening, pseudodrusen-like lesions.	YES	hyperreflective dots, vessel alterations
11	NO	normal	YES	LE: diffuse choroidal thickening	NO	normal
12	NO	vascular tortuosity	YES	LE: diffuse choroidal thickening	NO	hyperreflective dots
13	NO	no tessellation	NO	normal	NO	normal
14	NO	no tessellation	YES	RE: diffuse choroidal thickening	NO	normal
15	NO	no tessellation	YES	LE: diffuse choroidal thickening	NO	hyperreflective dots
16	NO	drusen-like lesion	YES	Bilateral: choroidal thickening	NO	hyperreflective dots
17	NO	no tessellation	YES	RE: diffuse choroidal thickening	NO	hyperreflective dots

NIR (near-infrared reflectance); SDOCT (spectral-domain optical coherence tomography); NA (not applicable); RE (right eye); LE (left eye).

**Table 3:** Enhanced depth imaging spectral-domain optical coherence tomography (EDI-SDOCT) choroidal thickness measurement in patients with Sturge–Weber syndrome. Measurements were performed manually from the inferior margin of Bruch’s membrane to the choroidoscleral junction.

Choroidal Thickness (μm) in the DCH Affected Eye	Choroidal Thickness (μm) in the Fellow Eye	Choroidal Thickness (μm) Inter Eye Difference
NA	341	NA
315	256	59
NA	202	NA
709	452	257
517	135	382
NA	NA	NA
NA	367	NA
454	233	221
520	270	250
814	366	448
1060	520	540
472	359	113
NA	345	NA
460	330	130
421	418	3
433	447	−14
462	368	94

NA (not applicable).

## Data Availability

The data presented in this study are available on request from the corresponding author.

## References

[1] Comi A.M. (2011). Presentation, Diagnosis, Pathophysiology, and Treatment of the Neurological Features of Sturge-Weber Syndrome. Neurologist.

[2] Shirley M.D., Tang H., Gallione C.J., Baugher J.D., Frelin L.P., Cohen B., North P.E., Marchuk D.A., Comi A.M., Pevsner J. (2013). Sturge-Weber Syndrome and Port-Wine Stains Caused by Somatic Mutation in GNAQ. N. Engl. J. Med..

[3] El Hachem M., Diociaiuti A., Galeotti A., Grussu F., Gusson E., Ferretti A., Marras C.E., Vecchio D., Cappelletti S., Severino M. (2025). Multidisciplinary, Multicenter Consensus for the Care of Patients Affected with Sturge-Weber Syndrome. Orphanet J. Rare Dis..

[4] Waelchli R., Aylett S.E., Robinson K., Chong W.K., Martinez A.E., Kinsler V.A. (2014). New Vascular Classification of Port-Wine Stains: Improving Prediction of Sturge-Weber Risk. Br. J. Dermatol..

[5] Lo W., Marchuk D.A., Ball K.L., Juhász C., Jordan L.C., Ewen J.B., Comi A. (2012). Brain Vascular Malformation Consortium National Sturge-Weber Syndrome Workgroup Updates and Future Horizons on the Understanding, Diagnosis, and Treatment of Sturge-Weber Syndrome Brain Involvement. Dev. Med. Child. Neurol..

[6] Ferretti A., Muscianese M., Fanfoni C., Bellone G., Mennini M., Di Nardo G., Abdolrahimzadeh S., De Marco G., Orsini A., Foiadelli T. (2024). Headache in Sturge-Weber Syndrome: A Systematic Review. Cephalalgia.

[7] Sabeti S., Ball K.L., Bhattacharya S.K., Bitrian E., Blieden L.S., Brandt J.D., Burkhart C., Chugani H.T., Falchek S.J., Jain B.G. (2021). Consensus Statement for the Management and Treatment of Sturge-Weber Syndrome: Neurology, Neuroimaging, and Ophthalmology Recommendations. Pediatr. Neurol..

[8] Bichsel C.A., Goss J., Alomari M., Alexandrescu S., Robb R., Smith L.E., Hochman M., Greene A.K., Bischoff J. (2019). Association of Somatic GNAQ Mutation with Capillary Malformations in a Case of Choroidal Hemangioma. JAMA Ophthalmol..

[9] Francis J.H., Milman T., Grossniklaus H., Albert D., Folberg R., Levitin G., Coupland S., Catalanotti F., Rabady D., Kandoth C. (2019). GNAQ Mutations in Diffuse and Solitary Choroidal Hemangiomas. Ophthalmology.

[10] Surve A., Azad S., Venkatesh P., Kumar V., Chawla R., Gupta V., Vohra R. (2019). Choroidal Vascular Pattern in Cases of Sturge-Weber Syndrome. Ophthalmol. Retin..

[11] Arora K.S., Quigley H.A., Comi A.M., Miller R.B., Jampel H.D. (2013). Increased Choroidal Thickness in Patients with Sturge-Weber Syndrome. JAMA Ophthalmol..

[12] Abdolrahimzadeh S., Scavella V., Battaglia D., Recupero S.M. (2016). Spectral Domain Optical Coherence Tomography of Choroidal and Outer Retinal Layer Thickness in the Sturge Weber Syndrome. Curr. Eye Res..

[13] Wu Y., Yu R.-J., Chen D., Xu L., Li M., Zhu L., Guo C.-Y., Guo W.-Y. (2017). Glaucoma in Patients with Eyes Close to Areas Affected by Port-Wine Stain Has Lateral and Gender Predilection. Chin. Med. J..

[14] Singh A.D., Rundle P.A., Vardy S.J., Rennie I.G. (2005). Photodynamic Therapy of Choroidal Haemangioma Associated with Sturge-Weber Syndrome. Eye.

[15] Schmidt-Erfurth U.M., Michels S., Kusserow C., Jurklies B., Augustin A.J. (2002). Photodynamic Therapy for Symptomatic Choroidal Hemangioma: Visual and Anatomic Results. Ophthalmology.

[16] Arepalli S., Shields C.L., Kaliki S., Emrich J., Komarnicky L., Shields J.A. (2013). Diffuse Choroidal Hemangioma Management with Plaque Radiotherapy in 5 Cases. Ophthalmology.

[17] Kubicka-Trząska A., Karska-Basta I., Oleksy P., Romanowska-Dixon B. (2015). Management of Diffuse Choroidal Hemangioma in Sturge-Weber Syndrome with Ruthenium-106 Plaque Radiotherapy. Graefes Arch. Clin. Exp. Ophthalmol..

[18] Azarcon C.P., Qiu R.L.J., Sobol E.K., Hubbard G.B., Craven C.M., Bergstrom C.S., Wells J.R. (2024). Iodine-125 Plaque Brachytherapy for Diffuse Choroidal Hemangioma. Retin. Cases Brief Rep..

[19] Puthussery J.C., Ufondu A., Cherian S., Singh A.D. (2025). Diffuse Choroidal Hemangioma: Ophthalmic Outcomes Following Intensity-Modulated Radiation Therapy. Taiwan. J. Ophthalmol..

[20] Thareja S., Lucero E., Ramasubramanian A. (2025). Choroidal Hemangioma Treatment with Propranolol—A Case Study in Sturge-Weber Syndrome and Systematic Literature Review. Semin. Ophthalmol..

[21] Thapa R., Shields C.L. (2013). Oral Propranolol Therapy for Management of Exudative Retinal Detachment from Diffuse Choroidal Hemangioma in Sturge-Weber Syndrome. Eur. J. Ophthalmol..

[22] El Mollayess G., Sleiman K., Ibrahim R., Bleik J. (2023). Intravitreal Aflibercept for Diffuse Choroidal Hemangioma in Sturge-Weber Syndrome. Middle East Afr. J. Ophthalmol..

[23] Anaya-Pava E.J., Saenz-Bocanegra C.H., Flores-Trejo A., Castro-Santana N.A. (2015). Diffuse Choroidal Hemangioma Associated with Exudative Retinal Detachment in a Sturge-Weber Syndrome Case: Photodynamic Therapy and Intravitreous Bevacizumab. Photodiagnosis Photodyn. Ther..

[24] Huang Y., Gangaputra S., Lee K.E., Narkar A.R., Klein R., Klein B.E.K., Meuer S.M., Danis R.P. (2012). Signal Quality Assessment of Retinal Optical Coherence Tomography Images. Invest. Ophthalmol. Vis. Sci..

[25] Gershoni A., Barayev E., Vainer I., Allon R., Yavnieli R., Shapira Y., Mimouni M., Geffen N., Nemet A.Y., Segal O. (2022). Thickness Measurements Taken with the Spectralis OCT Increase with Decreasing Signal Strength. BMC Ophthalmol..

[26] García Caride S., Fernández-Vigo J.I., Valverde-Megías A. (2023). Update on the Diagnosis and Treatment of Choroidal Hemangioma. Arch. Soc. Esp. Oftalmol..

[27] Shields C.L., Honavar S.G., Shields J.A., Cater J., Demirci H. (2001). Circumscribed Choroidal Hemangioma: Clinical Manifestations and Factors Predictive of Visual Outcome in 200 Consecutive Cases. Ophthalmology.

[28] Shields C.L., Atalay H.T., Wuthisiri W., Levin A.V., Lally S.E., Shields J.A. (2015). Sector Iris Hemangioma in Association with Diffuse Choroidal Hemangioma. J. Am. Assoc. Pediatr. Ophthalmol. Strabismus.

[29] Helmi H.A., Alkatan H.M., Al-Essa R.S., Aljudi T.W., Maktabi A.M.Y., Eberhart C.G. (2021). Choroidal Hemangioma in Sturge Weber Syndrome: Case Series with Confirmed Tissue Diagnosis. Int. J. Surg. Case Rep..

[30] Formisano M., di Pippo M.C., Scuderi L., Abdolrahimzadeh S. (2021). Current Concepts on Diffuse Choroidal Hemangioma in Sturge Weber Syndrome. Ophthalmic Genet..

[31] Ciancimino C., Di Pippo M., Rullo D., Ruggeri F., Grassi F., Scuderi G., Abdolrahimzadeh S. (2023). An Update on Multimodal Ophthalmological Imaging of Diffuse Choroidal Hemangioma in Sturge–Weber Syndrome. Vision.

[32] Yu Y.-Y., Li X.-X., Liang J.-H. (2020). Ruthenium-106 Plaque Brachytherapy for the Treatment of Diffuse Choroidal Hemangioma in Sturge-Weber Syndrome. Int. J. Ophthalmol..

[33] Callaway N.F., Mruthyunjaya P. (2019). Widefield Imaging of Retinal and Choroidal Tumors. Int. J. Retin. Vitr..

[34] Shields C.L., Shields J.A., Potter P.D. (1995). Patterns of Indocyanine Green Videoangiography of Choroidal Tumours. Br. J. Ophthalmol..

[35] Abdolrahimzadeh S., Parisi F., Mantelli F., Perdicchi A., Scuderi G. (2017). Retinal Pigment Epithelium-Photoreceptor Layer Alterations in a Patient with Sturge-Weber Syndrome with Diffuse Choroidal Hemangioma. Ophthalmic Genet..

[36] Tan C.S., Ouyang Y., Ruiz H., Sadda S.R. (2012). Diurnal Variation of Choroidal Thickness in Normal, Healthy Subjects Measured by Spectral Domain Optical Coherence Tomography. Investig. Ophthalmol. Vis. Sci..

[37] Moderiano D., Do M., Hobbs S., Lam V., Sarin S., Alonso-Caneiro D., Chakraborty R. (2019). Influence of the Time of Day on Axial Length and Choroidal Thickness Changes to Hyperopic and Myopic Defocus in Human Eyes. Exp. Eye Res..

[38] Abdolrahimzadeh S., Ciancimino C., Grassi F., Sordi E., Fragiotta S., Scuderi G. (2021). Near-Infrared Reflectance Imaging in Retinal Diseases Affecting Young Patients. J. Ophthalmol..

[39] Amirikia A., Scott I.U., Murray T.G. (2000). Bilateral Diffuse Choroidal Hemangiomas with Unilateral Facial Nevus Flammeus in Sturge–Weber Syndrome. Am. J. Ophthalmol..

[40] Venkataraman A., Al-Gilgawi A., Stoker I., Reddy M.A., Sagoo M.S. (2025). Ultrasound Guided Ru106 Plaque Brachytherapy for Treatment of Exudative Retinal Detachment in Children with Diffuse Choroidal Haemangioma. Eye.

